# Practical Medicine

**Published:** 1876-11

**Authors:** 


### II. Practical Medicine.

1. *Rheumatic Tetanus cured by Salicylic Acid.* Wunderlich. (*Arch. d. Heilkunde* XVII., 5.)

The excellent results obtained from the use of salicylic acids in cases of articular rheumatism, induced Dr. W. to give the remedy a trial in a case of tetanus of a rheumatic, not traumatic, origin. The patient had been thoroughly drenched, and in the course of one week the tetanic rigidity gradually extended over the muscles of the face, the neck, the back, the abdomen and the lower extremities. All these muscles were in a state of continuous contraction which could not be overcome by passive or active motions. The sensorium was clear; the sensibility of the skin was not altered; the respiration was regular, though superficial; pulse, slow; temperature, normal. The patient took half a gramme (7 grains) of salicylic acid every hour, which was rather difficult to administer, on account of the firmly closed jaws. After seventeen doses a marked improvement was noticed; the patient could open his mouth better, and the muscular contracture of the abdomen and back was less rigid. The acid was continued in the same dose every two hours, with a good effect on the relaxation of the muscles; but after the second batch of seventeen powders the patient had a ringing in the ear and a dullness of hearing to such an extent that the salicylic acid had to be discontinued. The following morning the patient was worse; the muscles of the whole body were rigid and painful; the respiration difficult. At once the acid was given again every two hours, and after the fifth dose the patient began to feel better, and continued to improve until he was perfectly well. He took 53½ grammes of salicylic acid within eighteen days.

2. *The Crepitant Râle — its Nature and Conditions of Production.* W. H. Workmam, M.D. (*Boston Med. and Surg. Jour.*, Aug. 3, 1876.)

If artificial respiration be made upon a cadaver which is free from disease, the respiratory sounds will be the same as in health.

If by removal of the chest walls and costal plura the lungs are allowed to collapse, artificial respiration gives a sound somewhat finer and dryer, but otherwise identical with the crepitant râle which is heard only during inspiration.

This sound depends upon two conditions: *First*, Abolition of vesicular spaces by contact of the vesicular walls; *Second*, entrance of air, suddenly separating walls and restoring spaces.

Physiology furnishes two examples of crepitant râle: *First*, During the first deep inspiration after one has lain quietly upon the back; and, *Second*, in the infant when the atelectatic lungs are aerated.

In 1872–3, Cornil and Graucher, Paris, injected melted tallow into the lung tissue through a small trocar, producing a solid nodule impermeable to air. Artificial respiration produced a crepitant râle in the neighborhood of this nodule, heard during or just at the end of each inspiration. A similar but still more marked result was produced when the lung was uncovered.

In the second experiment the tallow was injected into the pleural cavity, compressing the lung by a hard mass, at the circumference of which the râle was heard. The same was produced by the introduction of bits of wood into the fissure between the lobes of the lung.

These various observations show that the râle may be produced by various means, which compress the alveoli during expiration and allow their sudden expansion during inspiration.

Those diseases which produce this physical condition of the lung tissue at some stage, though varying greatly in their pathological condition, produce the râle at that stage of the disease.

If we consider the diseases in which the râle occurs, we find very diverse pathological conditions, but in all a condition analogous to the one mentioned.

In croupous pneumonia the râle is not heard during congestion, but after exudation has commenced, and certain portions have been solidified.

Hæmorrhagic infarction and tumors give a condition analagous to that of the first experiment, and here we have also well marked râles.

In pleuritis the compression is external to the lung, and, as in the case of the second experiment, we have râles, but only after pain has subsided.

Thus we find in all cases where the râle is heard that there is compression of the healthy lung tissue, bringing the walls of the air cells in contact, followed by inflation and separation of the walls. R. S. H.

3. *Diagnosis of the seat of lesion in Facial Paralysis.* S. G. WEBBER (*The Journal of Nervous and Mental Disease*, July, 1876.)

If the facial is paralyzed after giving off the chorda tympani, taste will, of course, be unaffected; if before that nerve separates, taste will be lost in the corresponding portion of the tongue. If before the stapedius branch is given off, the hearing becomes more acute. If at the point where the petrosal nerve arises, the sense of taste will be lost; there will be unnatural acuteness of hearing; the palate will deviate to one side. When the lesion is at the base of the brain, the sense of taste is preserved, but generally the palate deviates and there may be acuteness of hearing.

The general rule that when motor nerves are injured in their course, after their exit from the nucleus of origin, the muscles supplied by them lose their reaction to the faradic current, is also true of the facial nerve; hence, in all cases of severe lesion of that nerve, at the points above mentioned, the faradic reaction of the muscles of the face should be lost.

When there is complete paralysis of the facial branches, paresis of the palate, no disturbance of taste, simple diminution of electric irritability, and when unusual or decussated reflex action is present, lesion of the facial nucleus may be diagnosticated. One will be more certain that the lesion is seated in the bulb when the other nerves arising therefrom are also implicated (the hypoglossal, accessories, vagus, trigeminus, abducens).

Complete paralysis of the facial branches, paresis of the palate, retained reflex action, no disturbance of taste or hearing, normal electrical excitability, and paralysis of the opposite limbs show that the lesion is in the pons.

Partial paralysis of the facial branches, upper ones remaining free, paresis of the palate, retained reflex and electrical irritability, and paralysis of limbs on same side, leads to a diagnosis of the location of the lesion above the pons, in the crura cerebri and the cerebral hemispheres. Many times a paralysis of the motor oculi of the opposite side aids in fixing the seat of the lesion in the peduncle. D. R. B.

4. *Lesions in Paralysis Agitans.* B. F.* LAUTENBACH. (*The Journal of Nervous and Mental Disease*, July, 1876.)

The original impulse necessary to cause the movement of a set of muscles, originates in the peripheral gray matter of the hemispheres, and proceeds through the moter ganglia, the pedunculi cerebri, and then through the pyramidal fibres to the motor tract of the cord, from whence it proceeds along the necessary nerves to the muscles intended to be moved. The continuation of these movements depends upon the spinal cord. The regulation or co-ordination of these movements depends, not on the cerebellum, as has hitherto been supposed, but on the peripheral gray matter of the hemispheres.

The symptoms of paralysis agitans are due to an irritation of the regulating or co-ordinating fibres of the voluntary motor tract of the brain, which fibres pass from the peripheral gray matter of the cerebrum through the motor ganglia (probably through the nucleus caudatus), and the pedunculi cerebri to the cerebellum. D. R. B.

5. *A Case of Poisoning by Coal Gas.* R. DEXTER. (*The Journal of Mental and Nervous Diseases*, July, 1876.)

A servant girl retired, after closing her room, and, as she supposed, turned the gas off, but, in reality, leaving the burner open to the fullest extent. When found the next morning, she was in such a state of asphyxia as to render respiration feeble; frothing at the mouth; the surface of the body cold; pulse hardly preceptible at the wrist; sounds of the heart scarcely audible; she was entirely unconscious; pupils dilated to the fullest extent; no spontaneous movements; reflex activity diminished, but reflex action inducible by violent impressions. She was given fifteen drops of spirits of ammonia every ten minutes; after the second dose the pupils began to contract, the pulsė at the wrist improved and reflex movements beeame more active; in an hour and a half she was conscious, and spoke rationally; in an hour more was apparently well. D. R. B.

6. *Observations on Chronic Epilepsy.* A. MCLANE HAMILTON. (*The Journal of Mental and Nervous Diseases*, July, 1876.)

The first indication of treatment was to remove the cause if it could be ascertained. To allay erethism and reduce susceptibility of the medulla, and to administer some general nerve sedative, were the leading secondary indications. In those cases where there was tendency to anæmia the bromides did harm. The doctor claimed that no more than one drachm of either of the bromides should be administered during twenty-four hours. In those cases in which there was present a tendency to hyperæmia, ergot in large doses was recommended. Attacks of *petit mal* could be cut short by hypodermic injections of atropine. He regarded digitalis as a most important adjuvant in the treatment.

Nitrite of amyl was regarded as an agent which could afford temporary relief only, and was chiefly serviceable in those cases in which a succes sion of fits occured. The doctor regarded nitro-glycerine as an excellent prophylactic. He had used it in solution containing about one-quarter of a drop to five minims of alcohol, and had found it to produce almost an exact effect with nitrite of amyl, but the effects had been more permanent.

D. R. B.

7. *Diabetic Phymosis.* NIEPCE. (*Lyon Médical*, June 18, 1876.)

The author gives an interesting sketch, historical and pathological, of phymosis, as an epiphenomonon in cases of diabetes mellitus, illustrating his subject by an exceedingly instructive case. The suggestiveness of the whole paper will be seen in giving the author's second conclusion :

"If a phymosis occurs suddenly and spontaneously in a subject where diabetes has not been suspected, the urine should be analysed at once; while the secretion furnished by the parasitic balano-posthitis should be examined with the microscope. Curiously enough the phymosis is often the only symptom of the disease."

In the case treated by N. circumcision was actually performed before it was concluded to examine the urine; but when this last was done a flood of light was thrown upon the sudden and acute balanitis which had prostrated his patient, and the cause of which up to that time had been involved in the utmost obscurity.

8. *Congenital Hypertrophy of the Tongue, Relieved by Galvano-Cauterization.* VALERANI. (*Gaz. d. R. Accad. d. Med.*, June.)

The tongue of a child two months old had projected from the oral aperture to the extent of two centimetres since birth. The disfiguring enlargement interfered with the proper grasping of the mother's nipple, and induced a constant flow of saliva over the lips and chin. The tongue could not be crowded back into the mouth. All the extra-oral portion of the organ was surrounded with a platinum wire, and removed by galvano-cauterization without the loss of a drop of blood. The success was so pronounced that when cicatrization had occurred, no one would suspect, from an examination of the infant's mouth, the mutilation to which it had been subjected.

The following is Bizzozero's report of his microscopy of the excised segment:

"The microscope demonstrates that the macroglossia was due to simple hypertrophy of the tongue, in which the muscles, the connective tissue, the epithelium and all other normal constituents of the organ participated. A section of the portion removed could not be distinguished from one excised from a normal tongue, except in very moderate enlargement of the vessels and some of the capillaries distributed to the papillæ. There was no dilatation of the lymphatics, such as has been described in certain cases.

9 *Discordance of Pulse and Temperature in Typhoid Fever.* DESNOS. (*Jour. de Méd. et de Chir.*, July.)

Mistakes are often made respecting the intensity of typhoid fever, when the temperature is not taken in connection with examination of the pulse. D. treated a vigorous young man who, in the second week of typhoid fever, had but fifty-eight pulsations to the minute, when the temperature was registered at 39° C. His general condition was favorable though the typhoid condition was pronounced; tongue moist and intelligence unimpaired. Judging from the pulse and general phenomena only, the febrile condition would not have appeared severe. However, in this case, while the heart-beat increased to only ninety pulsations, the temperature rose to 40.5° C. Typhoid fever is one of those diseases which most frequently present this remarkable discordance. It is not rare to find the pulse not increased in frequency, while the temperature is very greatly augmented; though it is quite exceptional to note, as in this instance, a pulse below the normal standard. When there was complete defervescence in this patient, the number of pulsation per minute porportionately diminished. After several days of convalescence a relapse occurred in connection with neglect of hygiene, when the same disagreement recurred between the pulse and temperature record.

10. *Salicylic Acid in Microscopy.* EDWARDS. (*Amer. Jr. Microscopy*, June, 1876.)

The writer has kept casts of uriniferous tubules, from a case of nephritis, for two years, and in a perfect condition, in a solution of this acid. He has kept equally well, and for as long a time, leucorrhœal discharges, and remarks, "If salicylic acid will preserve such things, what will it not do." The strength of his solution is not stated, but it is probably a cold saturated solution in pure water.

11. *Neurotic Purpura.* TYRRELL. (*Pacific Med. and Surg. Jour.*, June, 1876.)

A school mistress, eighteen years old, had previously had ague, and had been mentally much worried. While menstruating she got wet to her skin in a shower. The menses ceased shortly, and next day she went to school feeling languid. In the afternoon she was seized, without premonition, with faintness, nausea and vomiting. Soon she had severe pain in the right knee and left ankle, and was unable to move. "Her legs, from the knee to the ankle, were covered with purpuric spots, varying in size from that of a pin's head to that of a quarter of a dollar." She was sure that in the morning no eruption existed here. She screamed from pain in the knee, which was swollen, and in the calves of the legs. Then she had cramps in the calves. She had warm fomentations and opiates to induce quiet. Then quinine and a cathartic were given, and in a few hours, and with the catharsis, the menses returned, and the eruption began to subside. On the third day it was all gone save the yellow stains, and the patient was well.

A stout man of forty-six, after severe exposure to cold and rain in a miasmatic swamp, had suddenly faintness, nausea, vomiting, palpitation and severe œsophageal spasms, with feelings of suffocation. He was cold and frightened. Soon there was reaction and fever, and then profuse dia phoresis. Quinine was given freely, but all fever did not abate till the fourth day, nor did the feelings of faintness and tightness in the chest disappear for a day or two.

On the abatement of fever there was excruciating pain in the knees and ankles, with puffiness and tenderness. This soon subsided, when a copious purpuric eruption appeared over the front of both legs, from the knees to the ankles. It felt like shot beneath the skin. In a day or two the eruption disappeared slowly, leaving the yellowish stains. Now the shoulders, elbows and wrists were successively attacked with excruciating pain. The purpuric rash came out upon the forearms, and the articular pain disappeared. In a few days this eruption was gone, and there was neuralgia over the left orbit, which was followed by conjunctivitis, "ecchymosis over the sclerous coat, epiphora, photophobia, and finally iritis," successively. No eruptions appeared, but there were swellings over either brow and in the neighborhood frequently, which were very evanescent. "As the pains and swellings left the eye, they returned with unexampled severity in the legs and knees, followed by a fresh eruption of purpuric spots." Muscular spasms became very frequent, affecting chiefly the flexors of the legs and arms. They usually only lasted a few hours, and were followed by a fresh eruption, which soon faded away.

Once he had orchitis, with œdema of the scrotum. The eruption never invaded the body, the neck or the face. There was no hæmorrhage from the gums, bowels, lungs or kidneys.

The disease resisted almost all medication. Large doses of quinine and iron would relieve it for a week or two, when suddenly the pains would return, to be followed by the eruption. A removal from a malarial region cured him at the end of six months. His general appearance had kept good throughout.

Another case was that of a man of forty, who had an erysipelatous inflammation over one eye from an injury. When this disorder was nearly well there occurred a chill, then terrific pains and cramps in the forearms and legs, which were followed shortly by the same kind of an eruption as that already described. Under antiperiodic treatment the man recovered in a few days. He had twice copious night sweats. There were no general symptoms of purpura. The man had lived in a malarial district.

Two other cases are reported, having essentially the same symptoms as those related — pains in the extremities, cramps of the most excruciating sort, and then the purpuric eruption; this disappearing to follow similar sufferings in a few days.

The patients had all resided where malaria affected many people. In none of them did the eruption attack the body, face or neck.

12. *Intermittent Diarrhœa.* ROTHE. (*Memoralilien*, 1876, 4.)

The doctor has repeatedly seen cases of diarrhœa attended by violent griping, and remarkable for the periodical regularity of the attacks. These cases resisted the usual treatment, but were quickly cured by a few doses of quinine. The most cases occurred in adults; still a few patients were children. The disease does not seem to be of a malarial nature, because they occurred in locations where intermittent fever or any other disorder due to malarial influences is unknown.

We will report one case for illustration: A robust, healthy woman, age twenty-five, consulted Dr. R. in September on account of diarrhœa, with violent, labor-like pains. Taking it for an ordinary intestinal catarrh, due to indigestion, he prescribed the necessary diet, and drops containing carbolic acid and iodine. After three days, no improvement; patient was ordered to bed, and acetate of lead, with rheum and opium, was given. After three days more the patient was worse, rather than better. She then informed the doctor of the singular fact that she would feel quite well during the day, until about seven o'clock in the evening. At that time regularly she would get the most violent pains in the stomach, followed by diarrhœic evacuations. These attacks she would get six to eight times in the night; but they would never return during the day. She took ten grains of quinine every three hours during one day, after which she passed a good and quiet night, and had no more attacks of periodic diarrhœa.

13. *Etherization of Young Children.* TRIPIER. (*Le Progrès Méd.*, No. 34.)

Hitherto this procedure has been esteemed harmless. Such is not the case; and Tripier reports three observations in which, although death did not actually occur, still the accidents were serious. The children were from five to eight years of age, and the point of interest in each was the sudden cessation of respiration, always accompanied by persistence of heart action. In one of these cases there was sudden arrest of respiration on three different occasions, and always in the same manner.

In no one of the cases did asphyxia occur, nor cyanosis. Syncope was excluded by the perceptible heart contractions. In all of the three cases there was abundant secretion of a more or less filiform mucus from the bronchi. Tripier inquires if the ether may not have provoked this secretion, and thus occasioned mechanical respiratory obstruction. With this idea in view, he has made some experiments upon cats from three to four weeks old. Now in all these cases he found at the outset of the etherization the respiration suddenly checked with the inspiratory movement — an accident which he never observed when chloroform was substituted for ether. Autopsies were made of all these etherized kittens, but in no case was there bronchial spume in the tubes. Therefore some other hypothesis must be framed to explain the occurrences in question. Possibly the action of the pneumogastric may be implicated. Henceforth, as he renounces ether, he proposes to employ chloroform instead.

Verneuil remarked that it was time to search actively for the causes of death in anesthesia. Two processes are only admitted at present — syncope and asphyxia. But the observations of Tripier, to which might be added many more, scattered here and there, show that a third mode must be admitted — still obscure and comparable to the action of arrest.

14. *Hygiene of the Hair.* Bazin. (*L'Union Méd. du Canada*, No. 5.)

The article prepared by the distinguished surgeon of the Saint Louis hospital for the *Dictionnaire des Sciences Médicales*, deserves the attention of all our readers:

The author commences by fully recognizing the action of those general influences which prove hurtful to the hair by inducing debility; that is to say, the general health must be established.

Under ordinary circumstances, the care required for the head should be directed merely to favoring the removal of the dust and deposit upon the hairy scalp.

In very young infants the brush and comb should give place to simple acetic or alkaline lotions, or the inunction of some fatty substance, such as cold cream or the oil of sweet almonds.

The practice of washing the scalp with warm or cold water is essentially bad, because it renders the hair dry, brittle and lusterless.

In women, the more or less complicated methods of dressing the head which prevail, necessitate the squeezing, dragging and twisting of the hairs in every direction — processes extremely unfavorable for their nutrition. Ladies should be taught that hairs, though insensible to pain, are not inert and lifeless, and that the most hygienic of all coiffures is that which leaves to the hair the greatest liberty and aeration and the most frequent repose.

The habit of wearing the hair long in men is bad, because they rarely spend the requisite time in cleansing it. The practice of clipping it close to the head is detestable, and absolutely contrary to the purpose for which it was designed.

Cutting the hair short in order to favor its growth, is the result of a prejudice which nothing can justify; while periodic hair cutting within reasonable limits is not injurious.

Contrary to the generally received opinion, Bazin concludes that the finest heads of hair are those which the scissors have never touched.

The habit of "refreshing" the hair, that is, of cutting away from time to time a small portion, may be indicated when the growth is thin, wasted or meagre.

The use of the razor should *always* be avoided, even when it is required to cut the hair very short, as in convalescence from grave disorders. Epilation, when employed for the purpose of removing white hairs, only hastens the supervention of canities.

The employment of cosmetics, instead of being allowed as of common usage, should be strictly confined to certain cases. Those who, when in perfect health, have naturally greasy hair, should be advised to use very weak alkaline lotions. Those, on the contrary, who have dry and harsh hair, may use oily applications.

Without expressing much confidence in the measures designed to prevent the loss of hair, the author concludes that sometimes the effort should be made.

Hair dyes are of two kinds. The first (galls, infusion of nux vomica, pomegranate,) is almost inoffensive, but gives uncertain and unstable results. The second, whose basis is generally lime, nitrate of silver, lead, or sulphate of iron, is successful in the result, but dangerous for employment.

